# Wait, can you remind me just why we need another journal focused on autophagy?

**DOI:** 10.1080/27694127.2022.2034253

**Published:** 2022-03-17

**Authors:** Daniel J. Klionsky, Fulvio Reggiori

**Affiliations:** 1 Life Sciences Institute, University of Michigan, Ann Arbor, MI, USA; 2 Department of Biomedical Sciences of Cells & Systems, University of Groningen, University Medical Center Groningen, Groningen, The Netherlands

**Keywords:** Acceptance rate, expansion, new journal, not all topics are equal, the bar to publish, we feel your pain

## Abstract

Well, because you ask that question, we are going to attempt to explain exactly why we do indeed need another journal focused on autophagy. If you are reading this far, you presumably know what “autophagy” means, so we do not have to impress upon you the importance of this topic, and how autophagic dysfunction is associated with numerous diseases in humans (okay, we felt compelled to slip that in anyway). Nor do we think that you need to be introduced to the journal *Autophagy*, which is just starting its eighteenth year and publishes papers on pretty much any topic; at least any topic that is connected to autophagy, which, after all, means pretty much any topic, if you get our drift. So, if *Autophagy* has done so well and serves such an important purpose, why do we need another journal? To find the answer, read on.

The number of papers published on the topic of autophagy has continued to increase over the past twenty years (Figure 1). Many, but certainly not all, of those papers have appeared in *Autophagy*, which is published both in print and online. The publishing world is certainly moving toward “online only” publications. At present, however, some readers still prefer to receive a hard copy. Such a requirement imposes certain restrictions. On the one hand, the publisher needs to plan the budget a year ahead and cannot simply accommodate a huge increase in manuscripts beyond the allotted number; there are specific costs associated with publishing including paper costs, formatting, and printing. On the other hand, there is a physical limit as to how large of a binding you can have on a journal, and we have pushed that limit with the last issues of volume 17 of *Autophagy. Autophagy Reports* (Figure 2) will be an open access (OA) journal without a specific page limit. Along these lines, one reason for launching *Autophagy Reports* is that more and more funding agencies are mandating that researchers publish in an OA journal.

Another issue is that as journals grow in stature, such as has been the case with *Autophagy*, they may see a rise in (okay, we are going to say this quickly, so we do not get into trouble by using this term) impact factor. One result is an increase in submissions. To maintain a high standing, the sheer increase in numbers means we need to become more selective about the papers that are accepted. What happens to those other papers? In this case, we are referring to “solid” papers that, for one reason or another, may not have a broad appeal to the journal readership. We consider it painful to turn away such manuscripts, and until now we have not had an alternative home for them. With the launching of *Autophagy Reports*, we can now provide another option — a journal that will maintain high standards, in which the editors and reviewers understand the importance of the topic, and the nuances of the research, in particular on autophagy-related pathways.

In addition, we know that not all papers are equal. Sometimes, a project does not have quite the outcome that we had predicted. Other times, the first author graduates or obtains an independent position. We have even heard that reviewers may, on rare occasions, request experiments that the authors consider to be too onerous or simply beyond the scope of the present analysis. Perhaps you would like to have a stronger or more complete paper, but a cost-benefit analysis indicates that additional work on that project is not warranted at the present time. Yet, you may be sitting on worthwhile data that would be of benefit to the autophagy community. If the data are solid, and the experiments were carried out using the appropriate methodology, we are in favor of publishing them. And now, we have the right home — *Autophagy Reports*.

*Autophagy Reports* will publish the following types of articles:

Research (basic science, translational and clinical)

Reviews

Brief Reports and Addenda

Resource

Commentaries and Views

Puncta

Meeting Reports

In addition to the editors-in-chief, *Autophagy Reports* has established an initial set of international section editors:

Jan Lünemann, University of Münster, Germany (immunology)

Sandra Maday, Perelman School of Medicine, University of Pennsylvania, Philadelphia, PA, US (neuroscience)

Michael J. Ragusa, Dartmouth College, Hanover, NH, USA (structural biology/biochemistry)

Sebastiano Sciarretta, University of Rome “La Sapienza”, Latina, Italy (cardiology)

Ann Simonsen, University of Oslo, Norway (cell biology)

We anticipate increasing the size of the editorial board and adding a group of associate editors. We also have an acquisitions editor, Adam Weiss, who helps to solicit puncta and advertise the journal.

We encourage you to consider *Autophagy Reports* as another option for publishing your autophagy research. We will set the same standards that have made *Autophagy* a reliable and trusted journal in our field of interest.

## Figures and Tables

**Figure 1:**
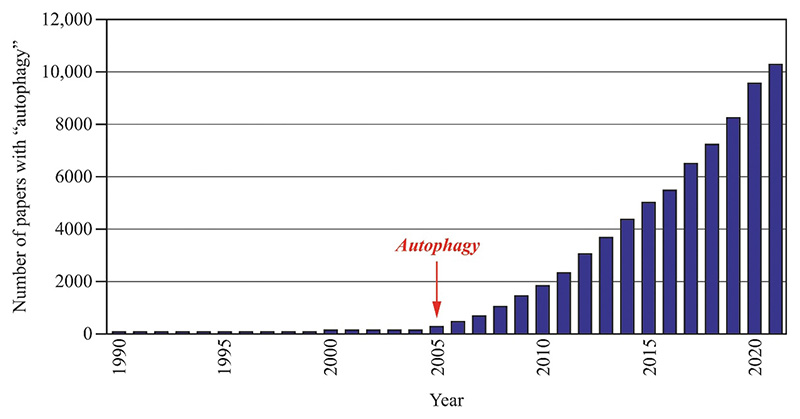
Graph illustrating the number of papers per year with the word “autophagy” in any field based on an EndNote PubMed search. The red arrow indicates the launch of the journal *Autophagy*.

**Figure 2:**
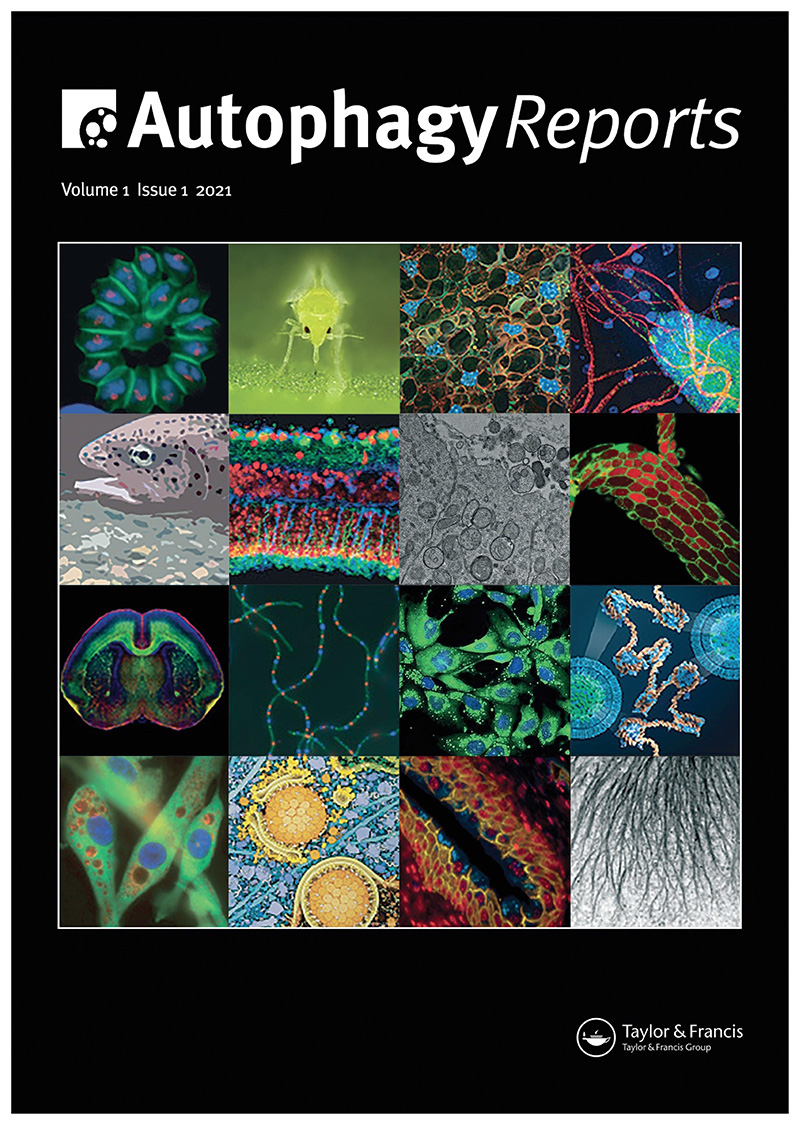
The cover of *Autophagy Reports* features a montage of images from previous covers of *Autophagy*.

